# The clinical features of pulmonary artery involvement in Takayasu arteritis and its relationship with ischemic heart diseases and infection

**DOI:** 10.1186/s13075-021-02675-9

**Published:** 2021-12-03

**Authors:** Hiroki Mukoyama, Mirei Shirakashi, Nozomi Tanaka, Takeshi Iwasaki, Toshiki Nakajima, Hideo Onizawa, Hideaki Tsuji, Koji Kitagori, Shuji Akizuki, Ran Nakashima, Kosaku Murakami, Masao Tanaka, Akio Morinobu, Hajime Yoshifuji

**Affiliations:** 1 Department of Rheumatology and Clinical Immunology, Graduate School of Medicine, Kyoto University, 54 Shogoin Kawahara-cho, Sakyo-ku, Kyoto, 606-8507 Japan; 2 Tazuke Kofukai, Medical Research Institute, Kitano Hospital, Osaka, Japan; 3 Department of Advanced Medicine for Rheumatic Diseases, Graduate School of Medicine, Kyoto University, Kyoto, Japan

**Keywords:** Takayasu arteritis, Pulmonary artery involvement, Ischemic heart diseases, Infection

## Abstract

**Background:**

Pulmonary artery involvement (PAI) in Takayasu arteritis (TAK) can lead to severe complications, but the relationship between the two has not been fully clarified.

**Methods:**

We retrospectively investigated 166 consecutive patients with TAK who attended Kyoto University Hospital from 1997 to 2018. The demographic data, clinical symptoms and signs, comorbidities, treatments, and imaging findings were compared between patients with and without PAI. TAK was diagnosed based on the American College of Rheumatology Classification Criteria (1990) or the Japanese Clinical Diagnostic Criteria (2008). PAI was identified using enhanced computed tomography, magnetic resonance imaging, or lung scintigraphy.

**Results:**

PAI was detected in 14.6% (*n* = 24) of total TAK patients. Dyspnea (25.0% vs. 8.6%; *p* = 0.043), pulmonary arterial hypertension (PAH) (16.7% vs. 0.0%; *p* < 0.001), ischemic heart disease (IHD) (29% vs. 9.3%; *p* = 0.018), respiratory infection (25.0% vs. 6.0%; *p* = 0.009), and nontuberculous mycobacteria (NTM) infection (20.8% vs. 0.8%; *p* < 0.001) were significantly more frequent, and renal artery stenosis (0% vs. 17%; *p* = 0.007) was significantly less frequent in TAK patients with PAI than in those without PAI. PAI and biologics were risk factors for NTM.

**Conclusions:**

TAK patients with PAI more frequently have dyspnea, PAH, IHD, and respiratory infection, including NTM, than TAK patients without PAI.

## Introduction

Takayasu arteritis (TAK) is an idiopathic vasculitis predominantly affecting the aorta and its branches. However, TAK can also affect the carotid, subclavian, renal, iliac, coronary, and pulmonary arteries (PA). Vessel wall inflammation leads to arterial wall thickening, dilation, stenosis, and occlusion. PA involvement (PAI) occurs in 5.7–25.93% of all cases [1–10]. TAK with PAI is occasionally complicated with pulmonary hypertension (PH), resulting in the risk of early mortality [8, 11]. The early detection of PAI in TAK patients is important for preventing disease progression. However, as the disease is rare, the previously reported data, including several cohort studies, remains insufficient, and the clinical features of TAK with PAI are not fully clarified. Therefore, we retrospectively investigated the clinical features of TAK with PAI.

### Patients and methods

The medical records of 186 consecutive patients with TAK who visited Kyoto University Hospital, Kyoto, Japan, from 1997 to 2018 were reviewed. Twenty patients were excluded due to insufficient information. The demographic data, clinical symptoms and signs, imaging findings, treatments, and comorbidities were reviewed and compared between TAK patients with and without PAI. This study was approved by the Ethics Committee of Kyoto University Graduate School and Faculty of Medicine (G412). All study procedures were performed following the Declaration of Helsinki principals. TAK was diagnosed based on the 1990 American College of Rheumatology Classification Criteria [12] or the 2008 Japanese Clinical Diagnostic Criteria [13]. PAI was screened using enhanced computed tomography (CT), magnetic resonance imaging (MRI), or lung scintigraphy and was defined as the presence of vascular involvement manifested as stenosis, occlusion, dilation, or aneurysm formation in either PA. Fever was defined as body temperature ≥ 38 °C. Hypertension was defined by systolic hypertension of ≥ 140 mmHg or diastolic hypertension of ≥ 90 mmHg. Blood pressure discrepancy between arms was defined as that of ≥ 10 mmHg. Ischemic heart disease (IHD) was defined as angina pectoris and myocardial infarction that could be confirmed on the medical record. All patients with pulmonary arterial hypertension (PAH) were diagnosed using right heart catheterization (RHC), based on a mean PA pressure of > 20 mmHg with a PA wedge pressure of ≤ 15 mmHg, a pulmonary vascular resistance of > 3.0 Wood Unit, and exclusion of other types of PH [14]. Six systemic artery involvement subtypes were classified considering the classification criteria proposed by Hata et al. (Types I, IIa, IIb, III, IV, and V) [15]. Serious infection was defined as viral, bacterial, or fungal infections requiring hospitalization or intravenous antibiotics. Tuberculosis and nontuberculous mycobacteria (NTM) infections were also considered serious infections because they can cause significant disability and sequela. Respiratory infection was defined as a serious infection of respiratory systems, including pneumonia and NTM pulmonary disease. NTM pulmonary disease was diagnosed using criteria proposed by the American Thoracic Society/Infectious Disease Society of America in 2007 [16]. All lung lesions including PAI and NTM were confirmed by radiologists.

Continuous variables were presented as means and standard deviations, and Student’s *t* test was used to compare the groups. Qualitative variables were presented as numbers and percentages, and Fisher’s exact test was used to compare the groups. Multivariate logistic regressions were used to detect factors independently associated with NTM infection. The data analysis was conducted using JMP ® 14 (SAS Institute Inc., Cary, NC, USA). Kaplan-Meier analysis was conducted using GraphPad Prism version 7.0b for Mac OS X, GraphPad Software, San Diego, CA, USA, www.graphpad.com. *P* values less than 0.05 were considered statistically significant.

## Results

**Fig. 1:**
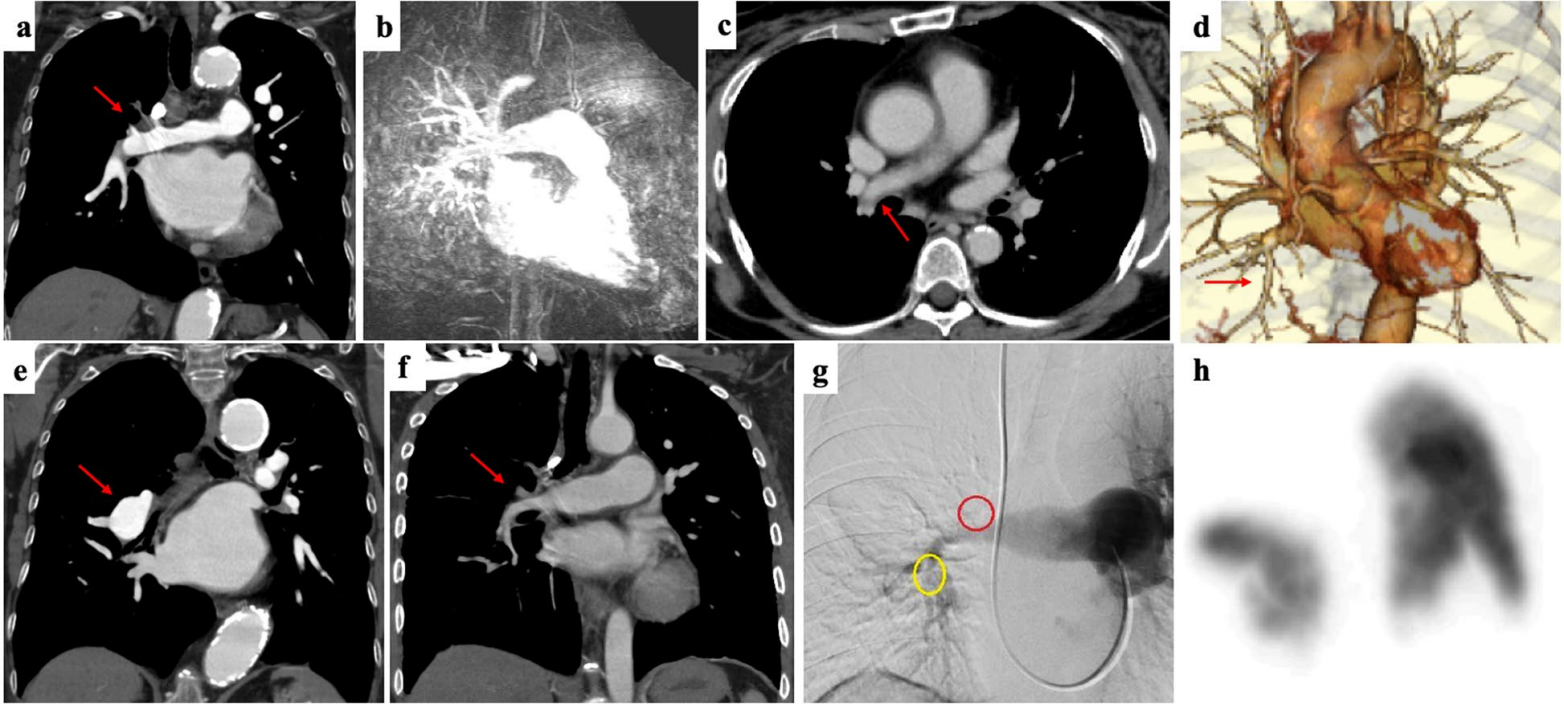
Representative pulmonary artery involvement images. Occlusion of the right upper pulmonary artery branches on enhanced computed tomography (CT) (**a**). Occlusion of the left main pulmonary artery on magnetic resonance angiography (**b**). The right main pulmonary artery vessel wall thickness (**c**) and stenosis of the lower pulmonary artery branches (**d**) on enhanced CT. Dilation of the right main pulmonary artery (**e**). Occlusion of the right pulmonary artery branches on enhanced CT (**f**) and angiography (**g**). A perfusion defect in the right lung (**h**)

**Fig. 2:**
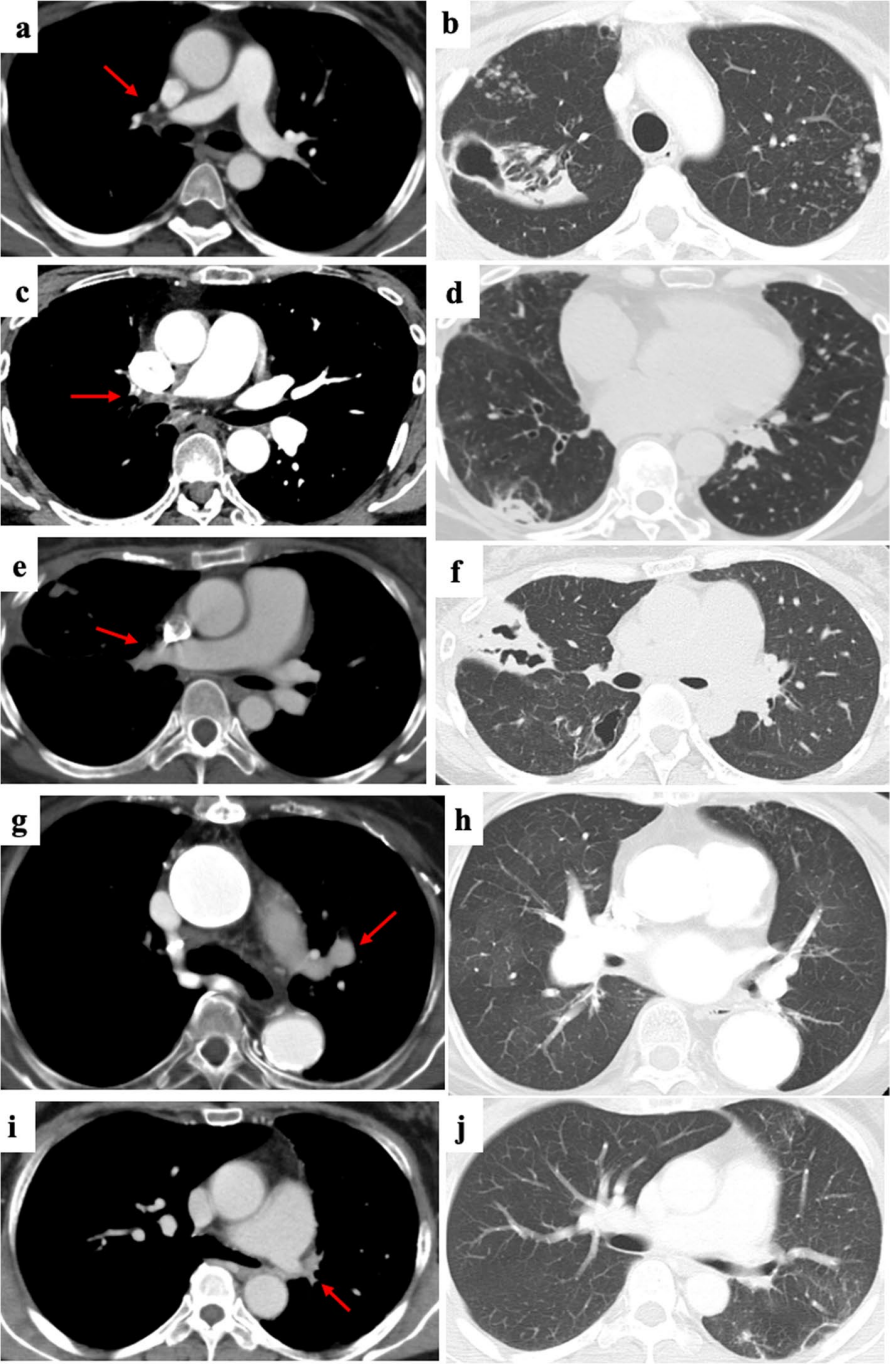
Pulmonary artery involvement (PAI) and nontuberculous mycobacteria (NTM) disease imaging on computed tomography. Patient 1: occlusion of the right upper and middle pulmonary arterial branches (**a**) and a large opacity with a cavity in the right lower lobe and ill-defined nodules in bilateral lungs (**b**). Patient 2: occlusion of the right main pulmonary artery (**c**) and a large opacity in the right lower lobe (**d**). Patient 3: occlusion of the right upper and middle pulmonary artery branches (**e**) and a large opacity with a cavity in the right lung (**f**). Patient 4: dilation of the left pulmonary artery branches (**g**) and ill-defined nodules in the left upper lobe (**h**). Patient 5: occlusion of the left main pulmonary artery before developing NTM disease (**i**) and ill-defined nodules in the left lung (**j**). The left and right images of patients 2, 3, and 4 were taken simultaneously, since the previous images were not available. Patient 6 did not have PAI. Therefore, the images are not presented

**Fig. 3:**
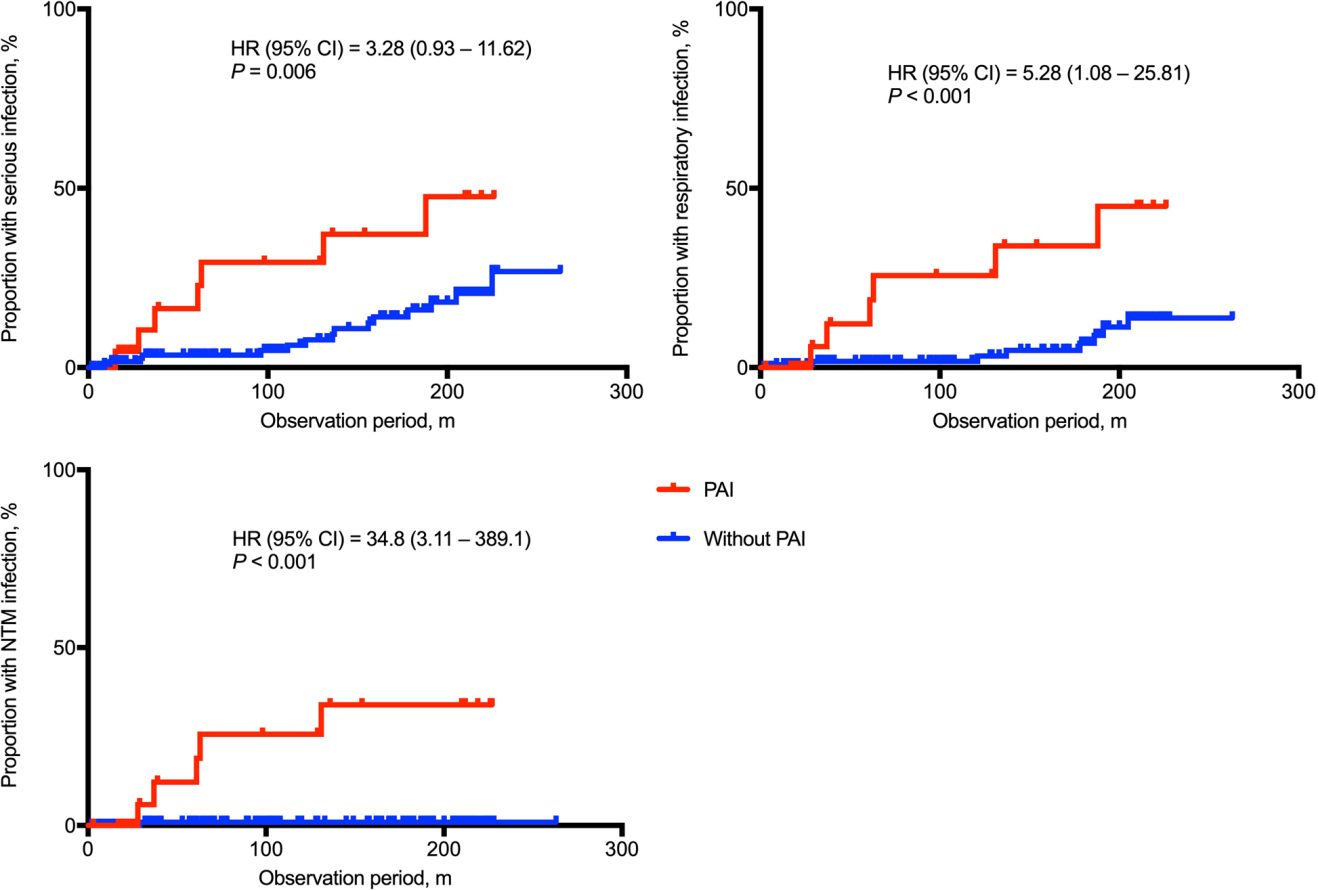
Serious infection, respiratory infection, and nontuberculous mycobacteria (NTM) infection during the observation period. Kaplan-Meier graphs show the rate of developing serious infection, respiratory infection, and NTM infection in Takayasu arteritis (TAK) patients with or without pulmonary artery involvement (PAI) during the observation period. The *y*-axis represents the cumulative incidence of infection. Hazard ratio (HR) represents comparison of survival curves (PAI/without PAI) using log-rank test. CI: confidence interval

Patients’ backgrounds and clinical characteristics are presented in Table 1; 166 TAK patients were included. PAI was detected in 14.6% (*n* = 24). Representative images of PAI are presented in Fig. 1. Of those with PAI, the mean age was 54.5 ± 17.0 years, duration from the onset was 20.7 ± 14.6 years, and the observation period was 7.8 ± 6.8 years, which did not differ from patients without PAI. Dyspnea (25.0% vs. 8.6%; *p* = 0.043), PAH (16.7% vs. 0.0%; *p* < 0.001), and IHD (28.6% vs. 9.2%; *p* = 0.022) were significantly more frequent, and renal artery stenosis (0.0% vs. 17.0%; *p* = 0.007) was significantly less frequent in TAK patients with PAI than in those without. Echocardiography was performed in all the TAK patients. In our hospital, RHC was performed when the peak tricuspid regurgitation velocity was 2.9 m/s or higher [17], as possible, if there were no contraindications. Based on this criterion, 8/166 patients underwent RHC and 4/8 were proven to have PAH. Respiratory infection (25.0% vs. 6.0%; *p* = 0.009) and NTM infection (20.8% vs. 0.8%; *p* < 0.001) were significantly more frequent in TAK patients with PAI than in those without. In this study, all patients with NTM infection had pulmonary NTM disease, and none had extra-pulmonary NTM disease. Further, all patients had received immunosuppressive treatment when the infection was diagnosed (Table 2). Representative images of patients with NTM infection are presented in Fig. 2. The predictors of NTM in patients with TAK were analyzed by univariate analysis with the Cox proportional-hazards model, which showed that PAI and biologics were risk factors for NTM (Table 3). Kaplan-Meier graphs depict the rate of developing serious infection, respiratory infection, and NTM infection in Fig. 3. Based on the log-rank test, TAK patients with PAI were at a significantly higher risk of these infection than those without.

**Table 1:** Patients’ characteristics and clinical symptoms

	PAI (*n* = 24)	Without PAI (*n* = 142)	*P* value
Patients’ characteristics			
Duration from onset (years)	20.7 ± 14.6	21.4 ± 15.6	0.835
Observation period (years)	7.8 ± 6.8	9.1 ± 6.8	0.355
Onset age	33.5 ± 13.1	30.1 ± 15.8	0.325
Age at surveillance	54.5 ± 17.0	52.2 ± 17.7	0.564
Female	22 (91.7%)	130 (91.6%)	1.000
Symptoms			
Systemic manifestation			
Fever	2 (9.5%)	33 (25.8%)	0.163
General fatigue	7 (33.3%)	54 (42.5%)	0.481
Hypertension	8 (38.1%)	53 (40.8%)	1.000
Head and neck			
Dizziness	8 (38.1%)	48 (37.2%)	1.000
Headache	7 (33.3%)	36 (27.9%)	0.610
Syncope	0 (0.0%)	8 (6.3%)	0.602
Eyes			
Eye symptoms	4 (20.0%)	26 (20.5%)	1.000
Visual disorder	2 (9.5%)	13 (10.1%)	1.000
Heart and lung			
Dyspnea	5 (25.0%)	11 (8.6%)	0.044
Chest compression	6 (30.0%)	16 (12.5%)	0.082
Hemosputum	1 (4.8%)	2 (1.6%)	0.368
Heart murmur	8 (40.0%)	40 (31.8%)	0.455
Ischemic heart disease	6 (28.6%)	12 (9.2%)	0.022
Aortic valve regurgitation	12 (57.1%)	55 (41.3%)	0.236
PAH	4 (16.7%)	0 (0.0%)	<0.001
Upper limbs			
Pulselessness	5 (23.8%)	31 (24.6%)	1.000
BP discrepancies between arms	5 (25.0%)	44 (34.1%)	0.610
Fatigue of upper limbs	5 (25.0%)	41 (32.3%)	0.611
Aorta			
Aortic aneurysm	1 (4.8%)	14 (10.9%)	0.696
Kidney			
Renal artery stenosis	0 (0.0%)	22 (18.5%)	0.043
Renal disorder	2 (9.5%)	18 (13.9%)	0.741
Infection			
Serious infection	7 (29.2%)	17 (12.7%)	0.059
Respiratory infection	6 (25.0%)	8 (6.0%)	0.009
Nontuberculous mycobacterium	5 (20.8%)	1 (0.8%)	<0.001
Treatment			
Glucocorticoid	21 (87.5%)	110 (77.5%)	0.416
Maximum glucocortioid (PSL)	31.1 ± 3.2	33.4 ± 1.4	0.508
Immunosuppressants	6 (26.1%)	54 (38.9%)	0.351
Biologics	5 (22.7%)	17 (12.2%)	0.206
GC only	11 (47.8%)	55 (39.6%)	0.455
GC + IS	3 (12.5%)	37 (26.1%)	0.200
GC + biologics	2 (8.7%)	1 (0.7%)	0.009
GC + IS + biologics	3 (13.0%)	15 (10.8%)	0.750
Antiplatelet therapy	11 (52.4%)	86 (68.8%)	0.210
Anticoagulation therapy	8 (38.1%)	21 (17.1%)	0.038
Cardiovascular surgery	2 (11.1%)	33 (25.8%)	0.242
Catheter treatment	3 (15.8%)	25 (21.2%)	0.764

Data are expressed as mean ± SD, or number (percentage). The difference of continuous variables was tested by Student’s *t* test, and that of qualitative variables was tested by Fisher’s exact test. *PAI* pulmonary artery involvement, *PAH* pulmonary arterial hypertension, *BP* blood pressure, *PSL* predonisolone (mg/day), *GC* glucocorticoid, *IS* immunosuppressants. The percentage is calculated as a ratio of the number of cases for which clinical information was available

**Table 2:** Clinical features and treatments of the patients complicate with NTM infection

Case	PAI	Age, gender	Species	Type	Treatment when infection was diagnosed	Maximum PSL dose	TAK disease duration (months) at diagnosis of NTM	Treatments for NTM
1	Yes	30F	*M. avium*	FC	PSL 15 mg	60	41	RFP, EB, CAM, SM, and operation
2	Yes	55F	*M. avium*	FC	PSL 7 mg, TCZ 162 mg s.c./week	40	241	RFP, EB, CAM
3	Yes	31F	*M. avium*	FC	PSL 5 mg, MTX 8 mg/w, TCZ 8 mg/kg DIV/4 weeks	20	71	RFP, EB, CAM, SM, and operation
4	Yes	72F	*M. intracellulare*	NB	PSL 2.5 mg	20	592	Observation
5	Yes	42F	*Not identified*	NB	PSL 12.5 mg	30	69	RFP, EB, CAM
6	No	64F	*M. intracellulare*	NB	PSL 5 mg	30	33	Observation

*PAI* pulmonary artery involvement, *NTM* nontuberculous mycobacteria, *PSL* predonisolone (mg/day), *TAK* Takayasu arteritis, *F* female, *M. avium* mycobacterium avium, *M. intracellulare* mycobacterium intracellulare, *FC* fibrocavitary type, *NB* nodular/bronchiectatic type, *PSL* prednisolone, *TCZ* tocilizumab, *MTX* methotrexate, *s.c* subcutaneous infusion, *DIV* drip infusion in vein, *RFP* rifampicin, *EB* ethambutol, *CAM* clarithromycin, *SM* streptomycin

**Table 3:** Predictors of NTM infection in patients with TAK (Univariate analysis)

Variables	HR (95% CI)	*P* value
Age	0.99 (0.94–1.04)	0.577
Duration from onset	0.99 (1.00–1.01)	0.104
Maximum PSL dose	1.00 (0.95–1.05)	0.982
Immunosuppressants	0.92 (0.17–5.00)	0.920
Biologics	8.44 (1.67–42.67)	0.010
PAI	34.86 (4.07–298.53)	0.001

Univariate analysis with the Cox proportional-Hazards model for the risk for NTM infection. *NTM* nontuberculous mycobacterium, *TAK* Takayasu arteritis, *PSL* predonisolone (mg/day), *PAI* pulmonary artery involvement, *HR* hazard ratio, *CI* confidence interval

PAI angiographic features are presented in Table 4. Right PA lesions were more frequent than the left. Occlusion was the most frequent type of PAI. The systemic artery subtypes [15] are presented in Table 5. In all patients and patients without PAI, Type V was the most frequent, followed by Type I. Types IIa and IIb were more frequent in patients with PAI than in those without but was statistically insignificant. Descending aorta involvement was less frequent in patients with PAI than patients without, and there were no Type IV patients or patients with renal artery stenosis with PAI.

**Table 4:** Angiographic features of PAI

Laterality	Right: 11 (45.8%), Left: 4 (16.7%), Bilateral: 9 (37.5%)
PA dilatation	3 (12.5%)
PA stenosis	8 (33.3%)
PA occlusion	13 (54.2%)
PA wall thickening	2 (8.3%)
Pulmonary thrombosis	2 (8.3%)

Data are expressed as numbers (percentage). *PA* pulmonary artery, *PAI* pulmonary artery involvement

**Table 5:** Classifications of vascular involvement

	Total (***n*** = 166)	PAI (*n* = 24)	Without PAI (*n* = 142)
I	37 (26.4%)	3 (14.3%)	34 (28.6%)
IIa	25 (17.9%)	7 (33.3%)	18 (15.1%)
IIb	14 (10.0%)	5 (23.8%)	9 (7.6%)
III	7 (5.0%)	1 (4.8%)	6 (5.0%)
IV	6 (4.3%)	0 (0.0%)	6 (5.0%)
V	51 (36.4%)	5 (23.8%)	46 (38.7%)

Data are expressed as numbers (percentage). *PAI* pulmonary artery involvement

Glucocorticoids were administered to 21 (87.5%) patients with PAI and 110 (77.5%) patients without PAI (Table 1; *p* = 0.416). Maximum glucocorticoid dose, immunosuppressant administration (40 patients were administered methotrexate, 27 azathioprine, 8 cyclophosphamide, 7 cyclosporines, 4 tacrolimus, and 1 mycophenolate mofetil), biologics administration (10 patients were administered tocilizumab, 7 infliximab, 3 ustekinumab, and 1 certolizumab pegol), a combination of the aforementioned therapies, antiplatelet therapy, cardiovascular surgery, and catheter treatment did not differ between the groups. Significantly more patients with PAI received anticoagulation therapy than patients without PAI (*n* = 8; 38.1% vs. *n* = 21; 17.1%; *p* = 0.038). Of those receiving anticoagulation therapy, 3 patients had deep venous thrombosis, 2 had atrial fibrillation, 1 had chronic heart failure, and 2 had only PAI. Patients who did not receive glucocorticoids, immunosuppressants, or biologics were considered to have an inactive disease.

One patient with PAI and seven without PAI died during the observation period. The patient with PAI died due to a urinary tract infection. The patients without PAI died due to pneumonia (*n* = 3), gastrointestinal bleeding (*n* = 2), carbon dioxide narcosis (*n* = 1), and sudden death with an unspecified cause (*n* = 1). Mortality did not differ between the groups based on Kaplan-Meier analysis (data not shown).

## Discussion

PAI is a rare TAK complication. The effect of PAI has not been fully elucidated. PAI is reported with TAK in 5.7 to 25.93% [1–10]. In this study, the presence of PAI was 14.6%, consistent with previous studies.

Our results indicated that TAK patients with PAI had dyspnea more frequently than those without PAI, consistent with the report by He et al. [7], suggesting that PAI should be considered when TAK patients complain of dyspnea. Further examination may find PAI sooner, potentially increasing the precautions taken for cardiovascular disease and respiratory infections, including NTM.

Wang et al. reported that right-sided PA lesions were more common than left-sided lesions in TAK patients with PH [18], and pulmonary arterial occlusions or near occlusions were found most frequently (88.9%), followed by pulmonary arterial stenosis (58.3%) and poststenotic dilation (27.8%). In this study, right-sided PAI was predominant, and occlusion (54.2%) was the most frequent finding, followed by stenosis (33.3%), and dilation (12.5%), similar to Wang et al.

Our results suggest that the TAK subgroups have different vascular involvement preferences. For example, Types IIa and IIb were more frequent in patients with PAI than in those without, descending aorta involvement was less frequent in patients with PAI, and no PAI patient had Type IV TAK. Gribbons et al. reported that patients with TAK and giant cell arteritis could be divided into six clusters [19], one of which was more likely to have abdominal lesions and another to have bilateral subclavian and carotid artery lesions.

TAK with PAI is often complicated with PH, resulting in a higher risk of early mortality [8, 11]. Mechanical obstruction of the pulmonary vasculature is a major cause of PH, and increased pulmonary vascular wall stiffness is also thought to be related [20]. In our study, PAH was a complication in 16.7% of TAK patients with PAI, and all PAH patients were diagnosed using RHC. This data regarding the PAH incidence in TAK patients is well-established because right heart overload detected by echocardiography does not always mean PAH. In our study, the presence of PAI did not lead to high mortality, perhaps because of the small sample size.

Using several imaging modalities to complement each other is preferable for diagnosis. First, traditional angiography was the gold standard for diagnosing TAK and detecting PAI [21, 22]. However, angiography is invasive and was replaced by CT and magnetic resonance angiography (MRA) [23]. CT and MRA also detect artery wall thickening [11, 24, 25]. Fluorodeoxyglucose-positron emission tomography is an even more sensitive modality to detect inflammation of artery, although it has lower power to detect lesions in small-diameter PAs [26, 27]. Pulmonary perfusion scintigraphy is also useful for PAI examinations. Perfusion defects indicate vascular occlusion, including small arteries that are sometimes not visible by CT [28–30]. Two patients were diagnosed with PAI by pulmonary perfusion scintigraphy in our study.

The treatment strategy for TAK patients with PAI is similar to that of TAK alone and includes immunosuppressive therapy, low-dose aspirin, and anticoagulative therapy. Reports suggest that the primary PAI treatment was immunosuppressive therapy [10]. Low-dose aspirin was used to prevent arterial ischemic events from TAK based on a single-center study that showed the efficacy of antiplatelet therapy [31]. Of the eight patients who received anticoagulation therapy, two patients received it for PAI and the others for deep venous thrombosis, atrial fibrillation, and chronic heart failure. No patients underwent PA stent implantation or surgery for PAs in the present study. Further research is needed to confirm the efficacy of antiplatelet or anticoagulant therapy for PAI.

To our knowledge, this is the first report to show an association between PAI and IHD. TAK may cause coronary artery stenosis, leading to myocardial infarction, heart failure, and sudden death [32]. It is reported that the coronary artery is affected in 10 to 60% of TAK patients [33–36]. Despite the high frequency of coronary artery lesions, only 5 to 20% of the cases are symptomatic [36], and most were detected by angiography or by cardiac MRI. Thus, the prevalence is likely to be underestimated. IHD in TAK patients is associated with a lower survival rate, and cardiac disease is the most chief cause of mortality in TAK patients [33, 37]. An older age at TAK onset, a longer TAK course, and a higher rate of traditional factors associated with atherosclerosis are reported IHD risk factors [38].. This study suggests that PAI is also an IHD risk factor.

Further, this study addressed the relationship between PAI and NTM infection. Sugiyama et al. reported a case of NTM disease concomitant with a PA occlusion caused by TAK, causing a pulmonary cavity lesion [39]. There were six reported pulmonary infarction cases with TAK, including two of cavitary lesions with chronic aspergillosis infections [40–43]. The etiology of pulmonary infections and cavitation is multifactorial. Destroyed lung construction, such as bronchiectasis, bronchiolitis, and interstitial lung disease, are known risk factors for pulmonary NTM disease [44]. Cases complicated with NTM infection and chronic thromboembolic pulmonary hypertension have also been reported [45]. A non-perfused lung may result in the poor delivery of inflammatory cells to the inflammation site [42, 45]. However, NTM infection itself causes cavitation. Therefore, it is difficult to clarify what develops first, pulmonary cavitation or NTM infection. In our study, all patients with NTM pulmonary disease received glucocorticoids before onset. Immunosuppressive treatment and poor lung perfusion are considered the main causes of NTM infection, but NTM infection sometimes renders patients with severe sequela, limiting the treatment options and making TAK treatment difficult. Thus, the early diagnosis of this complication is important.

This study has several limitations. This is a retrospective single-center study with a relatively small sample size. The observation periods differed among patients, and some were lacking data. Further, the NTM incidence rate may not apply to other countries due to racial and socioeconomic differences. A prospective multi-center study is needed to investigate the clinical features of TAK with PAI.

In conclusion, this study demonstrated that dyspnea, PAH, IHD, and respiratory infection, including NTM, were more frequently observed in TAK patients with PAI than in TAK patients without PAI. The early diagnosis and monitoring of PAI complications are required.

## Data Availability

The dataset supporting the conclusions of this article is included within the article.

## Abbreviations

CT: Computed tomography
IHD: Ischemic heart disease
MRI: Magnetic resonance imaging
NTM: Nontuberculous mycobacteria
PA: Pulmonary artery
PAH: Pulmonary arterial hypertension
PAI: Pulmonary artery involvement
PH: Pulmonary hypertension
RHC: Right heart catheterization
TAK: Takayasu arteritis
